# Diosgenin protects retinal pigment epithelial cells from inflammatory damage and oxidative stress induced by high glucose by activating *AMPK/Nrf2/HO‐1* pathway

**DOI:** 10.1002/iid3.698

**Published:** 2022-11-16

**Authors:** Yang Hao, Xuefeng Gao

**Affiliations:** ^1^ Department of Opthalmology The First Affiliated Hospital of Xi'an Jiaotong University Xi'an Shaanxi China; ^2^ College of Management Beijing Capital Normal University College of Management Beijing China

**Keywords:** AMPK/Nrf2/HO‐1 pathway, diosgenin, inflammatory damage, oxidative stress, retinal pigment epithelial cells

## Abstract

**Introduction:**

Diosgenin is a natural steroidal compound with reported antidiabetic and many other protective properties. This study aimed to explore the protective effect of diosgenin on high‐glucose (HG)‐induced retinal pigment epithelial cells.

**Methods:**

HG‐induced ARPE‐19 cells were considered as a cell model of diabetic retinopathy (DR). The viability and apoptosis of ARPE‐19 cells induced by HG treated with either diosgenin or Compound C (CC; dorsomorphin) were detected by Cell Counting Kit‐8 assay and flow cytometric analysis. The expression of apoptosis‐related proteins, inflammation‐related proteins, and *AMPK/Nrf2/HO‐1* pathway‐related proteins was detected by western blotting. The levels of inflammatory cytokines and detection of oxidative stress indexes were performed using the appropriate assay kits. The messenger RNA expression of inflammatory cytokines was detected by real‐time quantitative polymerase chain reaction.

**Results:**

There was no obvious effect of diosgenin on the viability of ARPE‐19 cells and the viability of ARPE‐19 cells was significantly reduced after HG induction. However, diosgenin increased the viability, inhibited the apoptosis, and reduced the inflammatory response and oxidative stress of ARPE‐19 cells induced by HG. In addition, diosgenin could activate the AMPK/Nrf2/HO‐1 pathway. CC, an AMPK inhibitor, could reverse the above changes caused by diosgenin treatment in ARPE‐19 cells induced by HG.

**Conclusions:**

Diosgenin could protect ARPE‐19 cells from inflammatory damage and oxidative stress induced by HG, by activating the AMPK/Nrf2/HO‐1 pathway.

## Graphical abstract

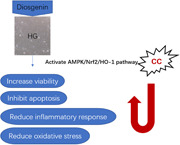

## 1 INTRODUCTION

Diabetic retinopathy (DR) is a common complication of diabetes mellitus and the main cause of vision loss in the elderly.^1^ With the increasing prevalence of obesity worldwide and the aging of diabetics, it is estimated that about 160 million people will be affected by DR by 2045.^2^ Patients with DR are usually asymptomatic in the early stage, but with the progression of the disease, patients will develop diabetic macular edema, vitreous hemorrhage, and traction retinal detachment, resulting in severe visual impairment.^3^ At present, the treatment of DR is mainly aimed at the vascular lesions caused by DR with limited efficacy^4^ and there is no effective treatment for the early neuropathy of DR.^5^

**Figure 1:**
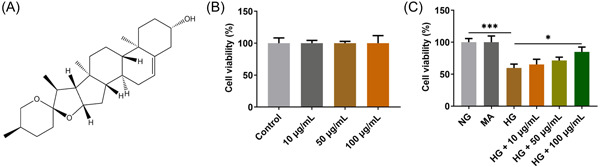
Diosgenin increased the viability of ARPE‐19 cells induced by high glucose (HG). (A) The chemical structure of diosgenin. (B) The viability of ARPE‐19 cells treated with different concentrations of diosgenin detected by Cell Counting Kit‐8 (CCK‐8) assay. (C) The viability of HG‐induced ARPE‐19 cells treated with different concentrations of diosgenin detected by CCK‐8 assay. *p < .05 and ***p < .001.

Diosgenin is a steroidal sapogenin obtained from, for example, *Solanum* and *Dioscorea* species. Its molecular structure is shown in Figure 1A. As an indispensable raw material for the production of steroidal hormone drugs, it has many pharmacological functions, including antitumor,^6^, ^7^ anti‐inflammatory,^8^, ^9^ and anti‐oxidation.^10^ It has also been shown to treat successfully cardiovascular diseases such as Type 2 diabetes and neurodegenerative diseases.^11^ Studies have shown that diosgenin can reduce cognitive impairment,^12^ aortic vascular dysfunction,^13^ and kidney damage in streptozotocin‐induced diabetic rats.^14^ Diosgenin can alleviate testicular injury in streptozotocin diabetic rats by reducing apoptotic oxidative stress and inflammation,^15^ and inhibits renal tubular epithelial fibrosis induced by high glucose (HG) through the epithelial–mesenchymal transition pathway.^16^ Diosgenin is an effective anticataract drug, which can not only significantly reduce the osmotic pressure of primary cultured lens epithelial cells induced by galactose in vitro, but also significantly delay the progression of galactose induced cataract in vivo in rats.^17^ However, the effect of diosgenin on DR has not been reported.

CTRP9 inhibits oxidative stress and apoptosis of retinal pigment epithelial cells induced by HG by activating the AMPK/Nrf2 signaling pathway. Knockout of GCN2 inhibits oxidative stress and apoptosis of ARPE‐19 cells induced by HG by activating the Nrf2/HO‐1 pathway. Therefore, we speculated that the AMPK/Nrf2/HO‐1 pathway was involved in the oxidative stress and apoptosis of retinal pigment epithelial cells induced by HG. Diosgenin can improve palmitic acid‐induced lipid accumulation in LO2 cells by activating AMPK/ACC/CPT‐1A and inhibiting the SREBP‐1c/FAS signaling pathway.^18^ Diosgenin can activate the catabolic pathway of skeletal muscle cells through AMPK and help to inhibit visceral fat accumulation.^19^ Diosgenin inhibits inflammation in an AMPK‐dependent manner, regulates the expression of adipokines in perivascular adipose tissue by regulating AMPK, and improves endothelial dysfunction.^20^ The above studies indicate that diosgenin can regulate the AMPK signaling pathway, but whether it also regulated the AMPK/Nrf2/HO‐1 pathway in retinal pigment epithelial cells induced by HG remains unknown.

In this study, we established a cell model of DR by HG‐induced inflammatory damage and oxidative stress in ARPE‐19 cells, and investigated the effect and underlying mechanism of diosgenin in HG‐induced ARPE‐19 cells.

## 2 MATERIALS AND METHODS

### 2.1 Cell culture

The retinal pigment epithelial cells (ARPE‐19 cells) were provided by the American Type Culture Collection (ATCC‐CRL‐2302). Cells were cultured in a humidified 5% CO_2_ atmosphere at 37°C in ATCC‐formulated DMEM: F12 Medium (cat. no. 30‐2006) containing 10% fetal bovine serum.

### 2.2 Cell treatment

ARPE‐19 cells were seeded in a 96‐well culture plate. After 12 h, cells were treated with diosgenin (10, 50, and 100 µg/ml) for 24 h, as in a previous study.^21^

ARPE‐19 cells were incubated with either normal glucose (5.5mM), HG (30 mM), or mannitol (MA, 30 mM) for 24 h. Subsequently, in the presence of HG, ARPE‐19 cells were treated with diosgenin (10, 50, and 100 µg/ml) for 24 h. Diosgenin was purchased from Aoke Biology Research Co. Ltd. ARPE‐19 cells were pretreated with AMPK inhibitor (Compound C [dorsomorphin], CC, 10 µM) for 2 h and then treated with HG for 24 h. In the presence of HG, ARPE‐19 cells were treated with diosgenin (100 µg/ml) for 24 h.

### 2.3 Cell Counting Kit‐8 (CCK‐8) assay

The viability of ARPE‐19 cells was detected by a CCK‐8 kit (CCK‐8; C0037, Beyotime). Briefly, ARPE‐19 cells were seeded into 96‐well plates at a density of 5 × 10^3^ cells/well. After the indicated treatment, 10 μl of CCK‐8 solution was added to each well, which was incubated for 2 h at 37°C. The absorbance was measured at 450 nm using the ELX‐800 microplate reader (BioTek).

### 2.4 Flow cytometric analysis

After the indicated treatment, the apoptosis of ARPE‐19 cells was detected using an Annexin V fluorescein isothiocyanate (FITC) and propidium iodide (PI) apoptosis detection kit (Keygen). Briefly, ARPE‐19 cells were collected and then added to 5 µl FITC and 5 µl PI for 15 min, avoiding light. Finally, the apoptosis of ARPE‐19 cells was observed by a flow cytometer (BD Biosciences).

### 2.5 Western blotting

After the indicated treatment, total protein from ARPE‐19 cells was extracted by RIPA (Beyotime) and detected by the BCA method (cat. no. P0010S; Beyotime), to determine the concentration. Then, equal amounts of protein (30 μg) were separated by sodium dodecyl‐sulfate polyacrylamide gel electrophoresis and then transferred to polyvinylidene difluoride (Beyotime) membranes. After sealing with 5% milk for 2 h, the membranes were incubated with primary antibodies against Bcl‐2 (ab32124; dilution, 1:1000; Abcam), Bax (ab32503; dilution, 1:1000; Abcam), cleaved caspase 3 (ab32042; dilution, 1:500; Abcam), COX‐2 (ab179800; dilution, 1:1000; Abcam), p65 (ab32536; dilution, 1:1000; Abcam), p‐AMPK (ab92701; dilution, 1:1000; Abcam), Nrf2 (ab62352; dilution, 1:1000; Abcam), HO‐1 (ab52947; dilution, 1:2000; Abcam), AMPK (ab271188; dilution, 1:1000; Abcam), and glyceraldehyde 3‐phosphate dehydrogenase (GAPDH) (ab9485; dilution, 1:2500; Abcam) overnight at 4°C. Goat antirabbit secondary antibody (ab6721; dilution, 1:2000; Abcam) was applied to incubate the membranes for another 1 h at room temperature. Protein bands were visualized using an enhanced chemiluminescence kit (Beyotime) and the intensity of the bands was analyzed using imageJ 1.51 software (National Institutes of Health).

### 2.6 Real‐time quantitative polymerase chain reaction (RT‐qPCR)

Total RNA was isolated using TRIzol (Invitrogen Inc.) and reversely transcripted to complementary DNA using a PrimeScript RT reagent Kit (Takara). RT‐qPCR was performed with a SYBR Green PCR kit (SY1020, Solarbio) using an ABI Prism 7500 sequence detection system (Applied Biosystems). The expression of tumor necrosis factor‐α (TNF‐α), interleukin (IL)‐6, and IL‐1β in the cell supernatant was calculated by the 2^−ΔΔCT^ method. *GAPDH* wasutilized as a reference gene. The primer sequences used are as follows: TNF‐α, forward 5′‐CTGGGCAGGTCTACTTTGGG‐3′ and reverse 5′‐CTGGAGGCCCCAGTTTGAAT‐3′; IL‐6, forward 5′‐GGTCCAGTTGCCTTCTCCCTG‐3′ and reverse 5′‐GCCCATGCTACATTTGCCG‐3′; IL‐1β, forward 5′‐TACCTGTCCTGCGTGTTGAA‐3′ and reverse 5′‐TCTTTGGGTAATTTTTGGGATCT‐3′; GAPDH, forward 5′‐AATGGGCAGCCGTTAGGAAA‐3′ and reverse 5′‐GCGCCCAATACGACCAAATC‐3′.

### 2.7 Enzyme‐linked immunosorbent (ELISA) assay

After the indicated treatment, the culture medium of ARPE‐19 cells was centrifuged to collect the supernatant. The levels of inflammatory cytokines, including TNF‐α, IL‐6, and IL‐1β in the supernatant, were measured by TNF‐α ELISA kit (cat. no. PT518; Beyotime), IL‐6 ELISA kit (cat. no. PI330; Beyotime), and IL‐1β ELISA kit (cat. no. PI305; Beyotime) according to the respective instructions of the manufacturer.

### 2.8 Detection of oxidative stress indexes

After the indicated treatment, ARPE‐19 cells were collected and washed with phosphate‐buffered saline at 4°C, two times. Then, ARPE‐19 cells were broken up by a homogenizer and centrifuged at 4°C to obtain the cell supernatant. The levels of oxidative stress indexes, including superoxide dismutase (SOD), glutathione peroxidase (GSH‐Px), and malonaldehyde (MDA in the cell supernatant, were measured by SOD (cat. no. A001‐3‐2), GSH‐Px (cat. no. A005‐1‐2), and MDA (cat. no. A003‐3‐1) assay kits from Nanjing Jiancheng Bioengineering Institute, according to the respective instructions of the manufacturer.

### 2.9 Statistical analysis

Data are expressed as mean ± SD. GraphPad Prism 8.0 software was used to conduct the statistical analysis using one‐way analysis of variance with Turkey's posthoc test, to analyze the values between different groups. *p* < .05 is considered statistically significant.

## 3 RESULTS

### 3.1 Diosgenin increased the viability of ARPE‐19 cells induced by HG

After ARPE‐19 cells were treated with different concentrations (10, 50, and 100 µg/ml) of diosgenin for 24 h, the viability of ARPE‐19 cells was not obviously changed (Figure 1B). The viability of the cells treated with 30 mM glucose (HG) was decreased compared with that in the control and MA groups. When the HG‐induced ARPE‐19 cells were treated with different concentrations (10, 50, and 100 µg/ml) of diosgenin for 24 h, the viability of ARPE‐19 cells in the HG + 100 µg/ml group was significantly increased compared with that in the HG group (Figure 1C).

### 3.2 Diosgenin inhibited apoptosis of ARPE‐19 cells induced by HG

**Figure 2:**
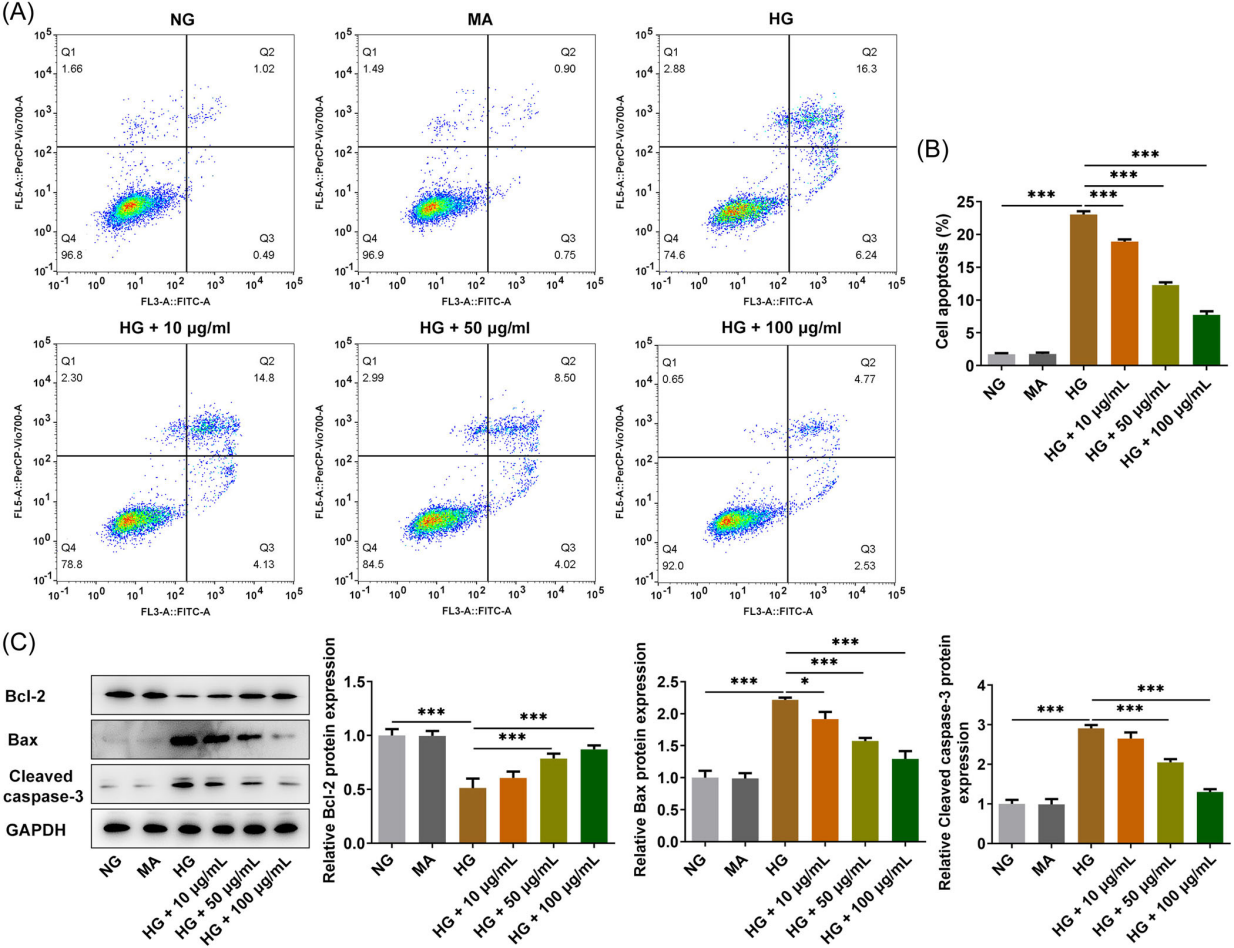
Diosgenin inhibited apoptosis of ARPE‐19 cells induced by high glucose (HG). (A, B) The apoptosis of HG‐induced ARPE‐19 cells treated with different concentrations of diosgenin was detected by flow cytometric analysis. (C) The expression of apoptosis‐related proteins in HG‐induced ARPE‐19 cells treated by different concentrations of diosgenin was analyzed by western blotting. *p < .05 and ***p < .001.

The apoptosis of ARPE‐19 cells was increased after HG induction; this was gradually suppressed by diosgenin from 10 to 100 µg/ml in a dose‐dependent manner (Figure 2A,B). The expression of Bcl‐2 was reduced and expression of Bax and cleaved caspase 3 was increased in ARPE‐19 cells of the HG group, but this could be reversed by diosgenin treatment (Figure 2C).

### 3.3 Diosgenin reduced the inflammatory response and oxidative stress of ARPE‐19 cells induced by HG

**Figure 3:**
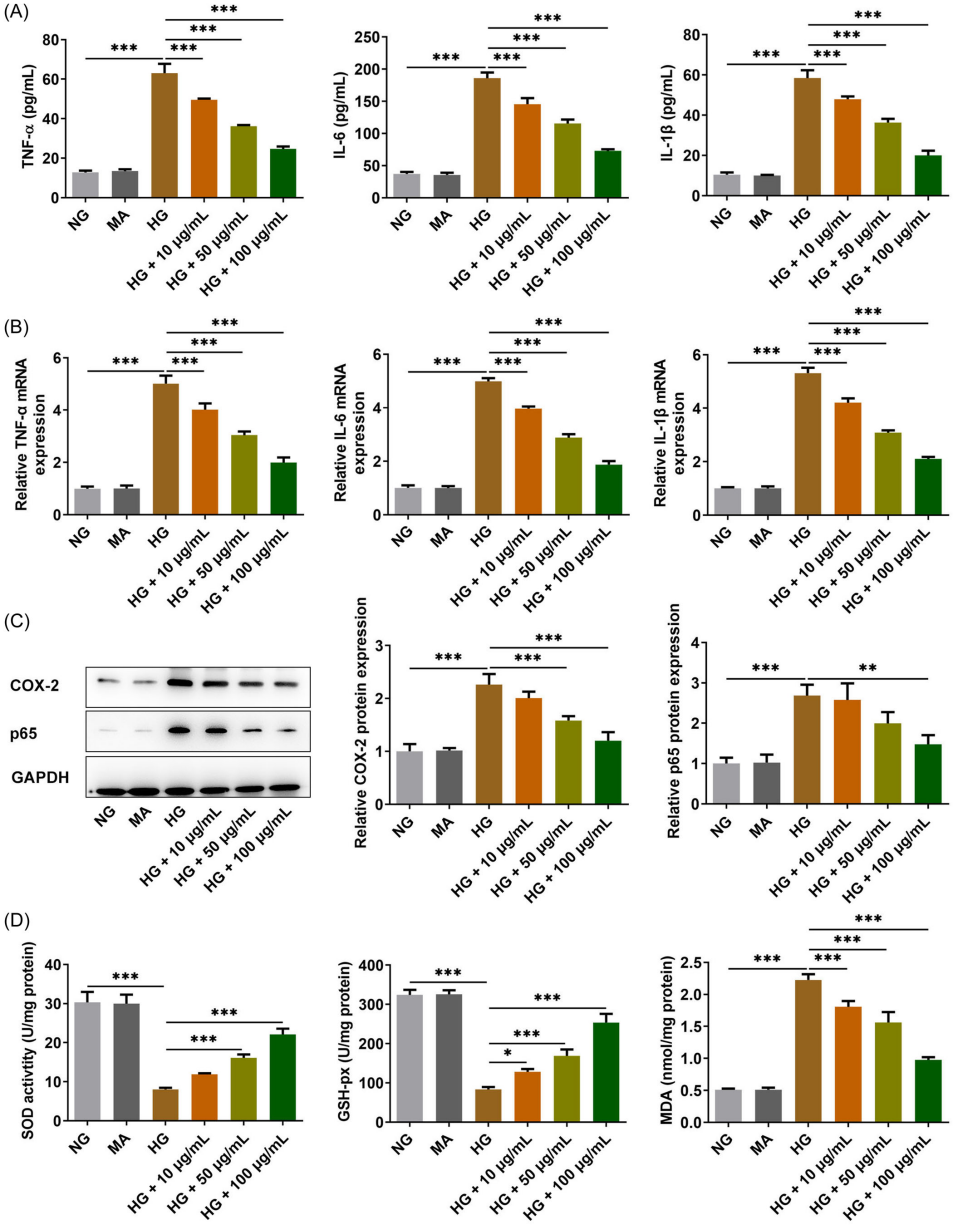
Diosgenin reduced the inflammatory response and oxidative stress of ARPE‐19 cells induced by high glucose (HG). The levels (A) and messenger RNA (mRNA) expression (B) of tumor necrosis factor‐α (TNF‐α), interleukin (IL)‐6, and IL‐1β in HG‐induced ARPE‐19 cells treated with different concentrations of diosgenin were detected by assay kits and real‐time polymerase chain reaction (RT‐PCR). (C) The expression of inflammation‐related proteins (COX‐2 and p65) in HG‐induced ARPE‐19 cells treated with different concentrations of diosgenin was analyzed by western blotting. (D) The levels of SOD, GSH‐Px, and MDA in HG‐induced ARPE‐19 cells treated by different concentrations of diosgenin was detected by assay kits. *p < .05, **p < .01 and ***p < .001.

The levels were enhanced of TNF‐α, IL‐6, and IL‐1β, as well as their RNA expression, in the culture medium of ARPE‐19 cells induced by HG, but decreased by diosgenin (Figure 3A,B). HG induction also promoted the expression of COX‐2 and p65, but this was downregulated by diosgenin in the HG group (Figure 3C). The levels of SOD and GSH‐Px were suppressed and MDA level was increased in ARPE‐19 cells of the HG group, but diosgenin could reverse these changes induced by HG (Figure 3D).

### 3.4 Diosgenin activated the AMPK/Nrf2/HO‐1 pathway

**Figure 4:**
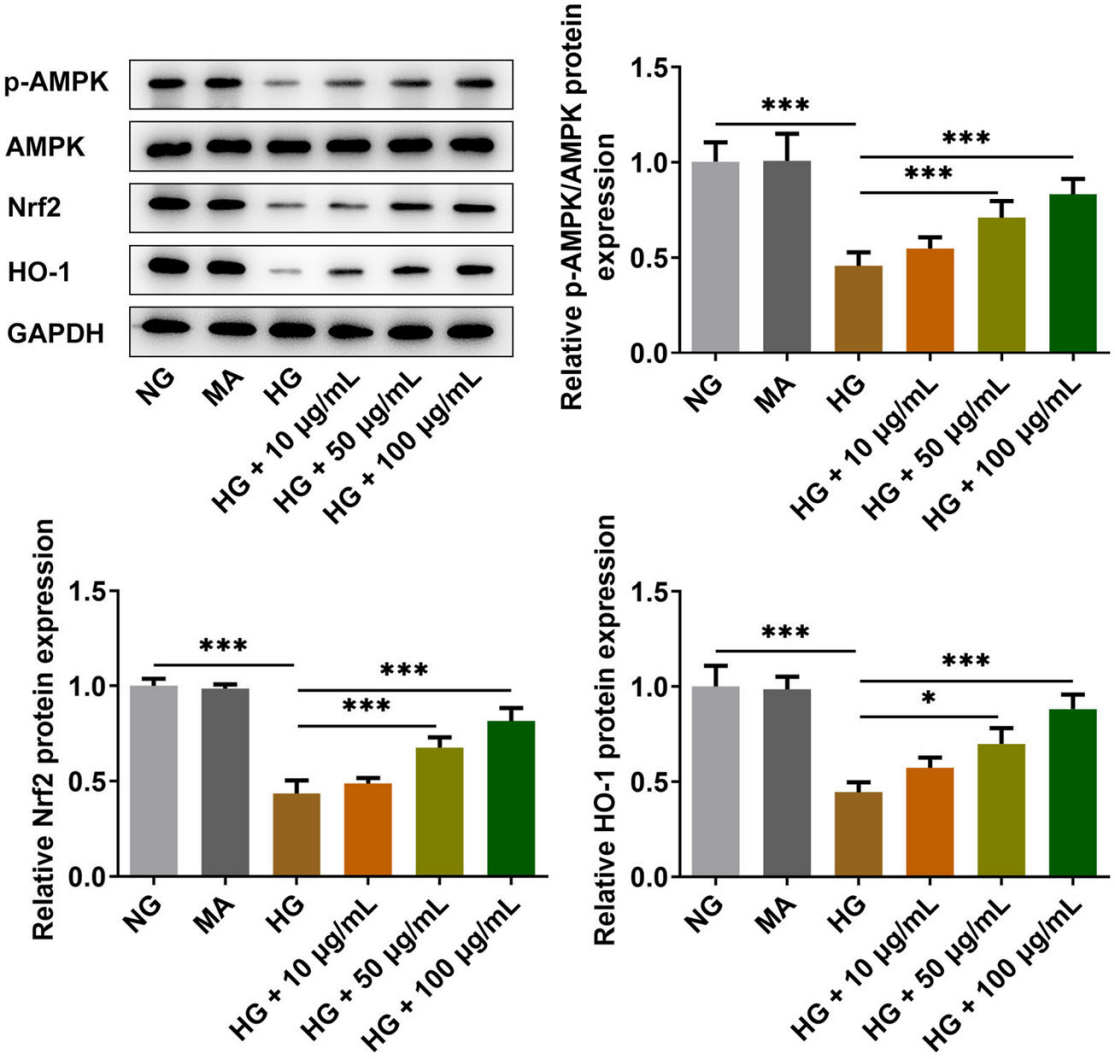
Diosgenin activated the AMPK/Nrf2/HO‐1 pathway. The expression of p‐AMPK, Nrf2, and HO‐1 in high‐glucose (HG)‐induced ARPE‐19 cells treated with different concentrations of diosgenin was analyzed by western blotting. *p < .05 and ***p < .001.

HG could suppress the expression of p‐AMPK/AMPK, Nrf2 and HO‐1 in ARPE‐19 cells of the HG group, but diosgenin could activate the above protein expressions in HG‐induced ARPE‐19 cells (Figure 4).

### 3.5 CC reversed the effect of diosgenin on the viability and apoptosis of ARPE‐19 cells induced by HG

**Figure 5:**
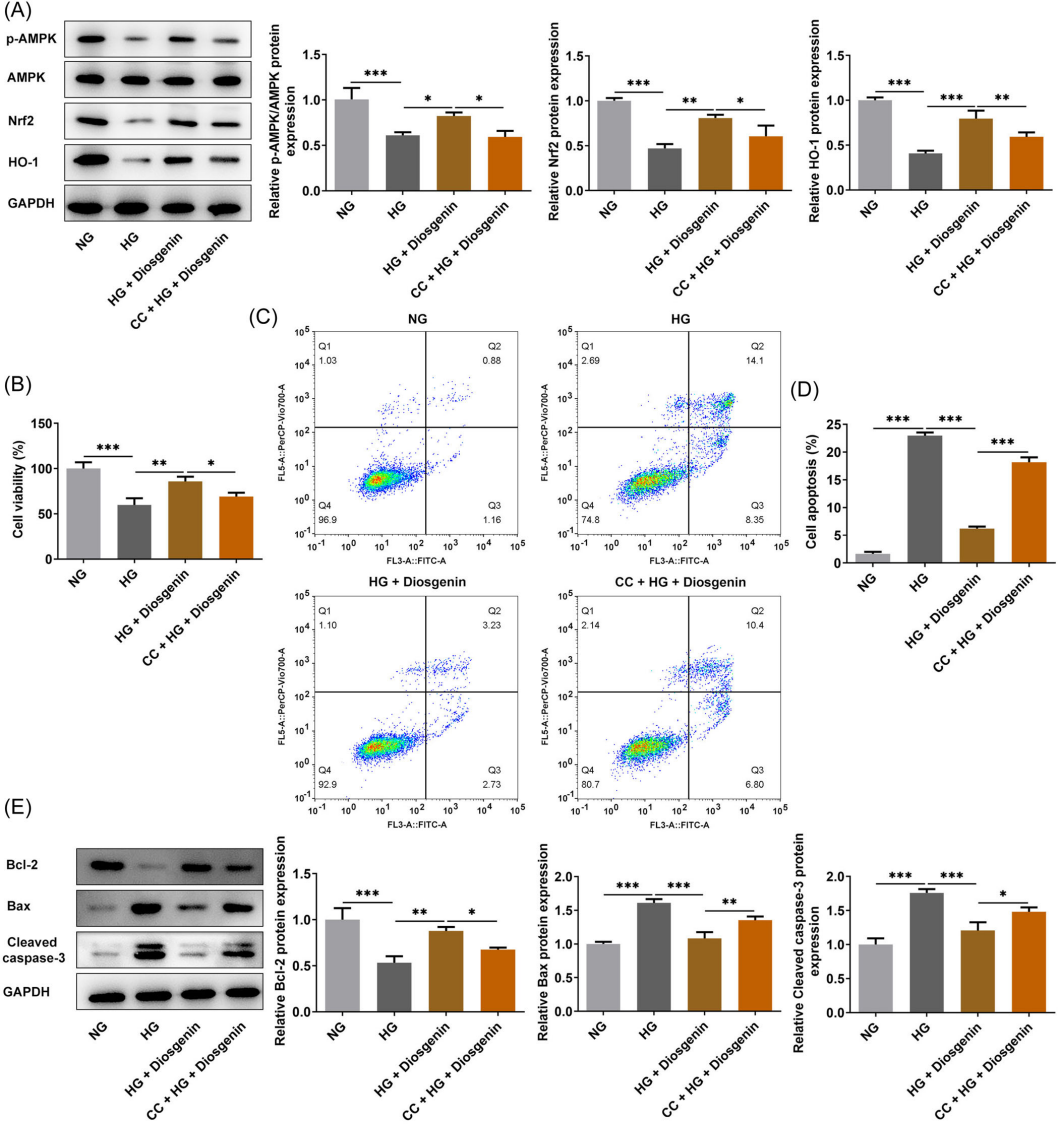
Compound C (CC) reversed the effect of diosgenin on the viability and apoptosis of ARPE‐19 cells induced by high glucose (HG). (A) The expression of p‐AMPK, Nrf2, and HO‐1 in HG‐induced ARPE‐19 cells treated with diosgenin; CC was analyzed by western blotting. (B) The viability of HG‐induced ARPE‐19 cells treated with diosgenin and CC was determined by Cell Counting Kit‐8 (CCK‐8) assay. (C, D) The apoptosis of HG‐induced ARPE‐19 cells treated with diosgenin and CC was detected by flow cytometric analysis. (E) The expression of apoptosis‐related proteins in HG‐induced ARPE‐19 cells treated with diosgenin and CC was analyzed by western blotting. *p < .05, **p < .01 and ***p < .001.

CC alleviated the promotion effect of diosgenin on the expression of p‐AMPK/AMPK, Nrf2, and HO‐1 in HG‐induced ARPE‐19 cells (Figure 5A). The viability was decreased (Figure 5B) and apoptosis was increased (Figure 5C,D) of ARPE‐19 cells in the HG + diosgenin group, which were pretreated with CC. Correspondingly, the expression of Bcl‐2 was downregulated and the expressions of Bax and cleaved caspase 3 were upregulated in ARPE‐19 cells treated with CC and diosgenin (Figure 5E).

### 3.6 CC reversed the effect of diosgenin on the inflammatory response and oxidative stress of ARPE‐19 cells induced by HG

**Figure 6:**
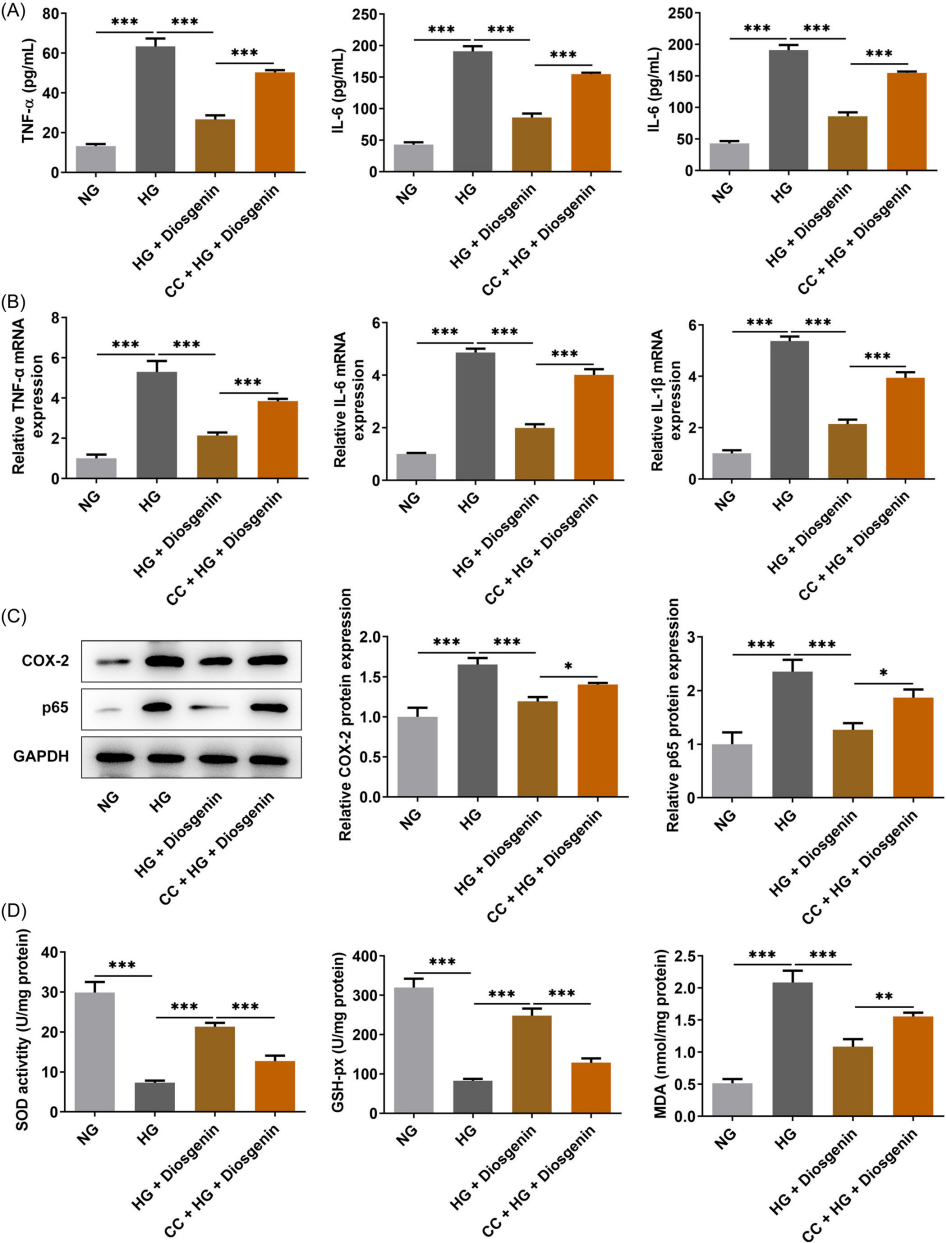
Compound C (CC) reversed the effect of diosgenin on the inflammatory response and oxidative stress of ARPE‐19 cells induced by high glucose (HG). The levels (A) and messenger RNA (mRNA) expression (B) of tumor necrosis factor‐α (TNF‐α), interleukin (IL)‐6, and IL‐1β in HG‐induced ARPE‐19 cells treated with diosgenin and CC were detected by assay kits and real‐time polymerase chain reaction (RT‐PCR). (C) The expression of inflammation‐related proteins (COX‐2 and p65) in HG‐induced ARPE‐19 cells treated with diosgenin and CC was analyzed by western blotting. (D) The levels of SOD, GSH‐Px, and MDA in HG‐induced ARPE‐19 cells treated with diosgenin and CC was detected by assay kits. *p < .05, **p < .01 and ***p < .001.

The levels and messenger RNA expression of TNF‐α, IL‐6, and IL‐1β were all upregulated in ARPE‐19 cells treated with CC and diosgenin (Figure 6A,B). The effect of diosgenin on the expression of COX‐2 and p65, and levels of SOD, GSH‐Px, and MDA were alleviated by CC in HG‐induced ARPE‐19 cells (Figure 6C,D).

## 4 DISCUSSION

Diosgenin, a steroidal sapogenin, largely exists as a glycoside in plants such as *Trigonella* (fenugreek), *Dioscorea* (yams), *Solanum* (nightshades), and *Polygonatum* species.^22^, ^23^, ^24^ Diosgenin has many biological activities, such as hypolipidemic, anti‐inflammatory, antiproliferative, hypoglycemic, and antioxidant.^25^, ^26^, ^27^ Streptozocin ‐induced diabetic rats treated with diosgenin showed reduced accumulation of serum creatinine and urine protein excretion, and an obviously decreased renal hypertrophy index, thereby improving renal function.^14^ Diosgenin promoted neurite outgrowth of PC12 cells, thereby improving diabetic neuropathy.^28^ Diosgenin decreased lipid deposition in the liver, suppressed the expression of lipogenic genes, and reduced the content of hepatic triglyceride in HepG2 cells, thus decreasing hepatic lipid content in diabetic mice.^29^ Therefore, we speculated that diosgenin might possess a protective effect on DR. The results of this study showed that diosgenin protected ARPE‐19 cells from inflammatory damage and oxidative stress induced by HG.

The balance between neurotrophic factors and inflammatory mediators in the retina of diabetic patients is disrupted, resulting in a chronic inflammatory response of retinal endothelial cells and nerve cells. Abnormal white blood cells interact with endothelial cells in an inflammatory environment, leading to damage to retinal blood vessels.^3^ The levels of inflammatory cytokines in diabetic patients are significantly increased, and their expression level is correlated with the severity of diabetes. At the same time, diabetics also have elevated levels of chemokines, which can cause retinal blood vessels to leak.^30^ This study also showed that the levels of TNF‐α, IL‐6, and IL‐1β were increased after HG induction in ARPE‐19 cells. Diosgenin could effectively suppress the upregulated levels of TNF‐α, IL‐6, and IL‐1β induced by HG in ARPE‐19 cells.

Cox‐2 is a membrane‐binding protein that plays an important role in mediating inflammatory responses. Overexpression of COX‐2 in cells can induce endothelial cell migration and tube formation, and thus participate in neovascularization. Animal experiments showed that COX‐2 protein was not only expressed in the retinal membrane of diabetic rats, but also increased with the prolongation of diabetic time, whereas no expression was found in the retina of normal rats.^31^ Resveratrol treatment suppressed the expression of nuclear factor‐κB (NF‐κB)–P65 and COX‐2 after renal ischemia/reperfusion injury in diabetic rats.^32^ Here, HG‐induced ARPE‐19 cells showed an increased expression of COX‐2 and p65, which was suppressed by diosgenin.

HG induction can upset the balance between oxidation and antioxidants, increasing levels of oxidation and reducing levels of antioxidants, leading to oxidative stress.^33^ Sheikpranbabu et al.^34^ found that pigment epithelium‐derived factor inhibited advanced glycation end‐induced reactive oxygen species (ROS) production, increased SOD and GSH levels, and prevented caspase‐3 activation through the analysis of porcine retinal pericells to decrease the pericyte loss in early DR disease. l‐carnitine could inhibit ROS and lipid peroxidation induced by HG and activate endogenous antioxidant components, including SOD and GSH‐Px, with antioxidant capacity.^35^ Quercetin could promote the expression of GSH, SOD, and catalase, inhibit the expression of NF‐κB and caspase‐3, and effectively prevent retinal neurodegeneration and oxidative stress damage in diabetic rats.^36^ In this study, we found that the levels of SOD and GSH‐Px were decreased while the MDA level was increased in HG‐induced ARPE‐19 cells. Consistent with the above studies, diosgenin protected against the oxidative stress damage in HG‐induced ARPE‐19 cells by upregulating the level of SOD and GSH‐Px, and downregulating the level of MDA.

AMPK directly catalyzed the phosphorylation of ser550 residues in Nrf2 protein to promote nuclear translocation of Nrf2 and increased the expression of downstream antioxidant proteins.^37^ In addition, AMPK could suppress oxidative stress through stimulation of the Nrf2‐dependent upregulation of HO‐1 and significant crosstalk has been observed in mammalian inflammatory systems^38^ and human endothelium.^39^ Kosuru et al.^40^ found that pterostilbene decreased cardiac oxidative stress and inflammation by activating the AMPK/Nrf2/HO‐1 pathway in fructose‐fed diabetic rats. Fibroblast growth factor 19 had an antioxidant effect in diabetic hearts by stimulating the AMPK/Nrf2/HO‐1 axis to protect the diabetic cardiomyocytes by alleviating the oxidative stress‐induced damage.^41^ Here, diosgenin was also found to activate the AMPK/Nrf2/HO‐1 pathway to weaken the inflammatory damage and oxidative stress in ARPE‐19 cells induced by HG. CC (an AMPK inhibitor) could reverse the effect of diosgenin on the inflammatory response and oxidative stress of ARPE‐19 cells induced by HG, which confirmed that diosgenin exerted its effects on ARPE‐19 cells through the AMPK/Nrf2/HO‐1 pathway.

In conclusion, it was found that diosgenin could promote viability and suppress apoptosis, inflammation, and oxidative stress of ARPE‐19 cells induced by HG through the activation of the AMPK/Nrf2/HO‐1 pathway, which was reversed by CC. The present study may provide a promising treatment of DR. There are also existing limitations. Animal experiments will be applied to investigate the treatment role of diosgenin in DR. Furthermore, the target genes of diosgenin to treat the DR will need to be studied.

## Data Availability

The data sets used and/or analyzed during the current study are available from the corresponding author on reasonable request.
